# Physical Conditions Essential to the Production of Tympanitic Resonance, and Pathological States of Pulmonary Tissues in Which It Occurs

**Published:** 1884-07

**Authors:** R. H. Babcock

**Affiliations:** Chicago


THE CHICAGO

### MEDICAL JOURNAL AND EXAMINER.

VOL. XLIX. JULY, 1884. NO. 1.

### ORIGINAL COMMUNICATIONS.

---

ARTICLE I.

### PHYSICAL CONDITIONS ESSENTIAL TO THE PRODUCTION OF TYMPANITIC RESONANCE, AND PATHOLOGICAL STATES OF PULMONARY TISSUES IN WHICH IT OCCURS. *By* R. H. BABCOCK, M.D., *Chicago.*

That deviation from the normal vesicular percussion note of healthy lung tissue, known as tympanitic resonance, is so significant a diagnostic sign, that I trust a few remarks upon the physical laws and pathological conditions favoring its development may not be out of place. Percussion derives its diagnostic import from the law of physics, that bodies, when struck, emit a sound whose quality, intensity, pitch, and duration are in accordance with the composition and molecular arrangement of the body struck. The quality of this sound is said to be sonorous or non-sonorous, according as the body emitting it does or does not contain air. The conditions of the human organism are such that, in percussing over its various internal viscera, we can only discriminate a difference in sound with relation to the presence or absence of air in the organs. Hence

1

the percussion note is either resonant or non-resonant, clear or dull. Yet it is highly probable that were the human ear sufficiently delicate in its sense-perception, it would be able to detect differences in the quality of the sounds emitted by the various viscera that are devoid of air; this difference in the quality of the sounds being dependent upon variations in the structure of the vibrating organs. Thus, the liver would give forth a sound of different quality from the spleen, that of the heart, again, differing from either of these. As it is, we only recognize a common character in the percussion note of these and other solid or airless viscera, viz., flatness.

But when we come to percuss over those which contain air within their walls, the stomach intestines, and respiratory organs, we are struck with the fact that the sound elicited is not always the same. The healthy lung tissue gives forth a tone peculiar to itself; and this "vesicular sound," as it is called, is very unlike the sound produced by striking the larynx, trachea, stomach, or bowels. The sound of these, in turn, differs in intensity, pitch, and duration, according to the organ whence it issues; yet it maintains a uniform quality which imparts to its individuality and whence its name, *tympanitic;* though to distinguish the locality of its production, it is severally called, laryngeal, tracheal, gastric and intestinal. Let us now inquire, What is it that gives this tympanitic note its individuality? Why should the air contained in the stomach, when set in vibration, emit a different tone from that of the lungs? Evidently it must depend upon conditions located in the enclosing walls that modify the character of the vibrations. The tympanitic sound, when analyzed with reference to its musical composition, is found to be made up of not merely one single tone, but of a keynote or "tonic" together with its over or secondary tones, whether these be "harmonics" or not; whereas, the normal vesicular note of the lungs is composed of but a single tone. Wintrich, who has investigated this subject most elaborately, found that when, by means of the acoustic flame, the vibrations of tympanitic and non-tympanitic sounds were compared, they displayed a striking contrast. The tympanitic sound consists of from six to ten regular vibrations that follow each other at regular intervals, increasing gradually in length until the maximum is reached, and then decreasing in the same manner.

The vibrations of the non-tympanitic sound, on the other hand, are seen to be irregular, both in height and succession.

But this knowledge of the character of its vibrations does not enable us to understand the physical conditions requisite to the generation of tympanitic resonance. Thanks to the painstaking experiments and observations of Wintrich, Gerhardt and others, chiefly Germans, we are able to formulate the following law, viz.: "A tympanitic note is produced when, by percussion, the column of air within a hollow cavity of regular form, and smooth not too tense walls, is set in vibration." (Gerhardt.) These conditions are found normally in the human body in the larynx, trachea, gastric, and intestinal cavities. Please notice that one of the essential conditions mentioned is, that the walls be *smooth* and *not* too tense, in other words, capable of reflecting vibrations. That smoothness of the walls is a prime essential to their reflecting ability, is well illustrated as follows: If percussion be practiced over a glass vessel which has been pressed down into a mass of snow, the sound elicited will be tympanitic. If the glass be now taken away, the cavity thus left will give a *non*-tympanitic note. This is because the walls of snow, unlike those of glass, are not smooth enough to reflect the soniferous vibrations; if the walls be very *tense*, they are *likewise* set in vibration by percussion, and themselves emit a tone, which obscures the tympanitic quality of the sound of the vibrating air. Therefore, according to Gerhardt, the lungs give out, under percussion, a non-tympanitic note, because of their too great tension. When partially relaxed under pathological conditions, or when taken out of the thoracic cavity, they sound, on the contrary, tympanitic. If the normal percussion note of the stomach and trachea be compared, the pitch of the latter will be found to be much higher.

Investigations have shown the *pitch* of the tympanitic sound to be subject to the following law, viz.: "The shorter the column of vibrating air, and the wider the opening with which the cavity communicates with the *outer* air, the higher the *pitch.*" (Gerhardt). This can be demonstrated by percussing the trachea and cheek, first with closed, then with open mouth.

Furthermore, it has been discovered that if a mass of foam, as of the white of egg beaten to a froth, be percussed, it will sound distinctly tympanitic, the pitch being directly proportionate to the size of the mass of bubbles set in vibration.

Likewise, a beer glass filled with the foam of ale or other malt liquor, will convey a veritable tympanitic percussion note to the ear of the experimenter. In this instance, also, the pitch follows the law laid down: the greater the bulk of foam percussed, the lower the tone, and conversely.

Bearing in mind, now, the conditions above stated as favoring the development of tympany, viz.: an air-containing chamber of regular form, whose walls are smooth and not too tense; and the admixture of air and liquid within a receptacle; and also considering the anatomical structure of the lungs and air tubes, together with the physical laws governing the transmission of vibrations,—keeping these things clearly in mind, we repeat, we shall be able to understand and interpret the morbid states of the pulmonary tissues in which we find tympanitic resonance on percussion. Let us now briefly consider them.

When a cavity is formed within the lungs, either by the dilatation of a bronchus, "bronchiectasis," or by destruction of the pulmonary tissue, through gangrene, abscess, or caseous degeneration, such cavity will give forth a tympanitic percussion note, if it comply with the conditions required. That is, if it have a regular outline and smooth walls, be occupied wholly or in part by air, and not too deeply located. If it communicate freely with a bronchus, the trachea, and thus with the outer air, its percussion sound will vary in pitch with the opening and closing of the mouth of the patient. The height of the sound emitted is also dependent upon the size of the cavity, or, in other words, upon the length of the vibrating column of air. Cavities are rarely round, but are generally longer than broad; hence, if they contain, in addition to air, liquid capable of shifting its location in response to the force of gravity, the diameter of the cavities will change accordingly, and thus affect the pitch of the percussion note. For example: if the long diameter of a cavity correspond with the antero-posterior axis of the body, and contain fluid, this liquid will occupy the floor of the cavity so long as the patient is standing or sitting, gravitating at once to the posterior extremity of it upon his assuming the dorsal decubitus. Now, the pitch of the percussion note over such a cavity is lower in the first instance and higher in the second. This is easy of explanation: in such a case, when the fluid fills the bottom of the cavity, and percussion is made over the anterior end of this latter, the vibrations traverse the entire length of the antero-posterior diameter of the cavity; when, on the other hand, the long diameter of the space is shortened, by the gravitation of the liquid to the posterior extremity, the vibration can only travel a shorter distance, and hence the pitch of the sound is raised. This alteration in the pitch of the tympanitic percussion note is called after Wintrich, who first directed attention to the phenomenon; and when this change of pitch is determined by variation in position it offers strong corroborative evidence of the presence of a cavity. It is not easy to determine the dimensions of the smallest cavity that is capable of generating tympany. It is often unmistakable in those of the size of a pigeon's egg, and may even be heard, under favorable circumstances, when the cavity is not larger than a hazel nut.

Another pathological condition favoring the production of tympanitic resonance, is the consolidation of a portion of lung surrounding a bronchus. Normally, the air within a bronchial tube, that has a tolerably direct communication with the trachea, is not thrown into soniferous vibrations by percussion, for the simple reason that those generated by percussion in the air of the pulmonary vesicles are dispersed and lost in traversing the alveolar network. But when the lung structure is changed by consolidation, it is rendered capable of transmitting with sufficient force the impulse imparted to it to create vibrations in the air contained in the broncho-tracheal tract; and this occasions a tympanitic sound, whose pitch rises and falls with the opening and shutting of the mouth (becoming lowest of all when both mouth and nose are closed), but remaining uninfluenced by the position of the patient's body. Its pitch, furthermore, corresponds to that of the broncho-tracheal respiratory sound, as determined by auscultation. As it is often extremely difficult to differentiate this condition of affairs from the existence of a deep-seated cavity, the effect of position upon the pitch of the tympanitic percussion note may be tested.

Since, as seems most probable, the normal percussion sound of healthy lungs is non-tympanitic by reason of the too great tension of these viscera, it follows that any condition of disease affecting their relaxation will favor the development of tympanitic resonance. Thus, in pleuritic effusions, we often detect this quality of the percussion sound immediately above the level of the fluid. Here the normal tension of the lung is overcome by the pressure of the effused liquid; and thus relaxed, the organ yields a tympanitic percussion note, whose pitch, as might be expected, does not change with the opening and closing of the mouth, since the air vibrating within the alveoli does not communicate directly enough with that in the trachea and larynx. When, however, the effusion is great, it is found, according to Gerhardt, that the pitch of the tympanitic note is higher in the upright than in the horizontal position. Moreover, this quality of the percussion sound above the level of the fluid is most evident anteriorly, near the sterno-clavicular articulation. In pneumo- and hydropneumo-thorax we obtain this same quality of sound; but here the explanation is different. To be sure, the lung relaxes its tension and retracts before the air entering the pleural cavity, whether its door of ingress be through a wound in the parietes, or a perforation, by ulceration, in the pulmonary pleura. But in this case the sound is generated by the vibrations of the column of air that intervenes between the chest wall and the retracted lung; and hence the impulsive force of the percussion shock fails to reach the relaxed pulmonary tissue. In pneumo-thorax the pitch is not influenced by change of position. Intra-thoracic tumors and a greatly enlarged heart may also so compress the lung as to favor the development of the tympanitic resonance in their immediate vicinity. Gerhardt also mentions as a clinical fact that, if a portion of a lung become greatly distended by an exudation, as in pneumonia, the adjacent parts undergo sufficient relaxation to give forth a tympanitic percussion note. I can not explain the mode of occurrence of this decreased tension of the parts surrounding a zone of consolidation, unless on the ground that the distended alveoli encroach upon those that have remained free, and by thus offering some impediment to the ingress of inspired air, interfere with the customary expansion of the pulmonary vesicles. And of course the natural tension of the lungs is dependent upon their normal expansion.

Finally, a condition which is capable of giving rise to tympany, and whose laws are not fully understood, is the presence of air and fluid at the same time within the pulmonary vesicles. This is easily demonstrated upon the cadaver. If water be injected into the cells of the lungs, yet not in sufficient quantity to expel all of the air, they will yield tympanitic resonance, which, as the pulmonary vesicles become entirely filled with liquid, gives place to flatness, and, on the other hand, returns to true vesicular resonance with the expulsion of the fluid. Now, a similar condition of things, viz., admixture of air and liquid, is present in œdema pulmonum, and in the first and third stages of pneumonia. At these times it is often possible to elicit a distinctly tympanitic quality of the percussion note. Therefore, in cases where it is not always easy to differentiate between pleuritis with slight effusion and the first stage of pneumonia, the detection of a faint though unmistakable tympanitic ring over the area of dullness, will serve to establish a diagnosis. Unfortunately, however, this quality is not always marked enough to be perceived, yet should be diligently sought for in doubtful cases. True tympanitic resonance in pulmonary emphysema is rare. Yet when it does occur it is difficult of explanation, and is probably dependent on some modifying circumstances not yet understood. In conclusion, let us consider, for a moment, one or two conditions, where, with the lungs perfectly healthy, the variety of resonance under discussion may be obtained in examination of the thorax. Occasionally, when the stomach or bowels are greatly distended with flatus and ride high up under the diaphragm, vigorous percussion over the base of the lungs will, in persons with elastic parietes, give tympanitic resonance; this is readily explained by the fact that the impulse imparted to the chest walls is conducted to the flatulent viscera and sets their contents in vibration. The same cause operates when the "cracked metal sound" is sometimes elicited by the forcible percussion of the chest of a young child suffering with bronchitis, a condition where cavities can manifestly not exist. Again: Friedrich, in his work on diseases of the heart, narrates the case of a woman who was ill with pericarditis. A few hours prior to her death a high-pitched tympanitic percussion note was elicited over the præcordia, and at the autopsy the pericardium was found distended with fluid and gas. Such instances are of extreme rarity.

